# Direct anterior approach for open correction of Slipped Capital Femoral Epiphysiolysis: surgical technique and clinical outcome

**DOI:** 10.1016/j.jcot.2026.103572

**Published:** 2026-07-20

**Authors:** Frans-Jozef Vandeputte, Siebe Clarebout, Abner Sergooris, Mathieu Gevers, Frank Van Praet, Kristoff Corten

**Affiliations:** a Department of Orthopedic Surgery and Traumatology, Ziekenhuis Oost Limburg (ZOL) Genk, Synaps Park 1, 3600, Genk, Belgium; b European Hip Center, Boskant 18, 2260, Westerlo, Belgium; c Faculty of Medicine and Life Sciences, Hasselt University, Diepenbeek, Belgium; d REVAL, Rehabilitation Research, Faculty of Rehabilitation Sciences, Hasselt University, Diepenbeek, Belgium

**Keywords:** Slipped capital femoral Epiphyses, SCFE, Direct anterior approach

## Abstract

**Background:**

Slipped Capital Femoral Epiphysis (SCFE) is an idiopathic hip disorder in adolescents. With or without surgery, avascular necrosis (AVN) is the most dangerous complication. For open reduction and internal fixation, the modified Dunn procedure is considered the gold standard. This study presents an open epiphyseal realignment through the Hueter-interval and reports on (1) surgical technique of direct anterior approach (DAA) based surgical realignment, (2) radiographic correction of biomechanics, and (3) AVN-incidence after surgery.

**Methods:**

A single-center retrospective case series was conducted including all patients who underwent open realignment for SCFE via the direct anterior approach between January 2014 and January 2025. Pre- and postoperative Southwick and alpha angles were measured by two independent observers. Complications, including avascular necrosis, chondrolysis, and degenerative changes, were assessed at final follow-up. Descriptive statistics were used. Interobserver agreement of radiographic measurements was assessed using the intraclass correlation coefficient (ICC).

**Results:**

Twelve patients (13 hips; 9 boys and 3 girls) were included. Median age and BMI were respectively 12.9 years old (IQR 12.2-13.7) and 23.1 kg/m^2^ (IQR 19.9-29.5) kg/m^2^. One patient showed bilateral involvement. Median preoperative AP - and lateral Southwick angle were respectively 117.5 (IQR 95.8–137.3) and 59.6 (IQR 35.1–71.8) degrees. After open realignment, median postoperative AP – and lateral Southwick angles were respectively 139.5 (IQR 135.0–143.3) and 14.7 (IQR 11.3–20.9). Thus, a correction of the lateral Southwick angle of 31.2 (IQR 17.4–54.2) was achieved. Follow-up was 6.0 years (IQR 2.4-8.3) and none of the patients developed AVN. Median Alpha-angle on Dunn view at final follow-up was 44.5 (IQR 42.8–68.1).

**Conclusion:**

The DAA described in this series, can be considered a reproducible technique for an open realignment of displaced SCFE. None of the cases had an AVN and the approach does not appear to limit correction potential.

**Level of evidence:**

Level 4 Therapeutic study.

## 1 Background

Slipped Capital Femoral Epiphysis (SCFE) represents the most prevalent hip pathology in the adolescent population.^1^ It typically manifests between 9 and 16 years of age, with a peak incidence around 12 years, and more frequently in boys than girls.^2^ The epiphysis, imprisoned in the acetabulum, rotates around the epiphyseal tubercle resulting in anterior displacement and external rotation of the metaphysis.^3^^,^^4^ The etiology of SCFE is considered multifactorial and contralateral involvement can present with a delay.^5^ Metabolic and endocrine abnormalities are biological risk factors, while obesity and femoral neck retroversion are the most recognized biomechanical contributors.^5^^,^^6^ Histological studies reveal altered chondrocyte metabolism within the proximal femoral physis, leading to extracellular matrix changes.^7^^,^^8^ Clinically, SCFE is categorized as stable or unstable, based on weight-bearing ability.^9^^,^^10^ Symptom duration distinguishes acute-, acute-on-chronic, and chronic forms. Slip-severity (mild, moderate or severe) is assessed using the Southwick angle.^10^

In-situ fixation has traditionally been the standard treatment. However, a displaced SCFE results in abnormal hip biomechanics, increasing the risk of early secondary degenerative changes to the hip joint.^11^ Nowadays, in-situ fixation is reserved for mild slips, while moderate and severe cases prefer an open realignment procedure.^12^^,^^13^

The Dunn procedure, later modified by Ganz using surgical hip dislocation and a trochanteric flip osteotomy in a posterior approach, is widely regarded as the reference technique for anatomical capital realignment in unstable or severe SCFE, with superior deformity correction and a favorable long-term outcome compared with in-situ fixation.^11^^,^^14^, ^15^, ^16^, ^17^ However, the modified Dunn procedure uses a posterior approach to achieve anatomical realignment at the cost of a surgical hip dislocation and trochanteric osteotomy, with associated approach-related morbidity and a prolonged period of protected rehabilitation.^18^

Parsch et al. corrected the slip deformity through an anterolateral approach with an anterior-based subcapital trapezoidal osteotomy, avoiding surgical hip dislocation and trochanter osteotomy.^19^ The same osteotomy can be performed through the Hueter-interval.^20^^,^^21^ However, this technique does not restore the epiphysis onto its original metaphyseal bed and is not a true anatomical capital realignment.^19^, ^20^, ^21^, ^22^

This study presents an open epiphyseal realignment through the Hueter-interval in a direct anterior approach (DAA). It reports on (1) surgical technique of DAA-based surgical realignment, (2) radiographic correction of biomechanics, and (3) AVN-incidence after surgery.

## 2 Methods

### 2.1 Patients

This was a single center retrospective case series, performed at the department of Orthopaedics and Traumatology at X. Ethical approval for this study was obtained from the institutional review board at X (protocol number IRB-Z-2025128). No patient/guardian consent was needed for this retrospective study. All patients who underwent open realignment of SCFE through the DAA between January 2014 and January 2025 were included. Cases with previous open reduction procedures were excluded. Surgical procedures were executed by two surgeons. Electronic patient records with the clinical and surgical notes were reviewed.

### 2.2 Clinical and radiological evaluation

All acute, or acute-on-chronic patients were admitted through the emergency department. In these patients, a physical examination was limited due to pain and discomfort. Chronic cases were evaluated during outpatient consultation by an orthopedic surgeon. Operative duration and length of hospital stay were noted. Following surgery, wound healing and discomfort due to hardware was monitored.

Pre- and postoperative anteroposterior (AP) pelvic and Lauenstein radiographs were available for all patients. Radiographic assessment was performed at the routine follow-up visits at 6 weeks and 3 months postoperatively. From one year postoperatively onward, follow-up consisted of anteroposterior pelvic and Dunn views. Radiographs were analyzed by two independent observers, and the mean of their measurements was used for analysis. A SCFE was scored mild, moderate or severe, using the slip angle on a Southwick AP and lateral method as described by Lindell et al.^23^ The Alpha angle was measured on Dunn views as described by Ekhtiari et al.^24^ At final follow-up, all X-rays were evaluated for signs of AVN, chondrolysis and degenerative changes of the joint. In addition, hip length discrepancy was measured on anteroposterior pelvic radiographs as the side-to-side difference in the perpendicular distance from the inter-teardrop line to the most prominent proximomedial point of the lesser trochanter. No magnification correction was applied because calibration markers were not consistently available.

### 2.3 Surgical procedure

Weight-adjusted intravenous cefazolin prophylaxis was administered before starting general anesthesia. The DAA on a standard table through a bikini incision was used, with careful preservation of the ascending branches of the lateral circumflex femoral artery (LCFA). In all cases, the vessels were intact at the end of the procedure. A medially based U-shaped capsulotomy provided exposure of the femoral neck, preserving vascular supply to the capsule.^25^

Following vascular assessment of the cephalad arteries, a T-shape incision of the metaphyseal periosteum enabled subperiosteal dissection to visualize the anterior physis (Fig. 1.1). The physis and the posterior callus was opened with gentle blows on a curved osteotome; this was initiated in the middle of the physis, then inferiorly and finally superiorly. The physis was removed to minimize traction on the cephalad vessels and the posterior callus was fragmented (Fig. 1.2). Once the metaphysis was freed, slight lateral traction was applied with a Schanz-screw in the greater trochanter through a lateral incision. The curved osteotome was introduced into the metaphyseal-epiphyseal fissure in a reverse orientation, with its convex surface against the metaphysis and its tip directed toward the epiphysis. The osteotome was used as a lever to guide the metaphysis posteroinferiorly into internal rotation while bringing the epiphysis anteriorly into external rotation. The maneuver was repeated gently until acceptable reduction was achieved, with continuous attention to the cephalad retinacular vessels. If required, limited superior bone removal was performed to keep the vessels relaxed. In selected cases, slight hip flexion and distal traction on the greater trochanter helped redirect the metaphysis into valgus and internal rotation beneath the epiphysis.

Reduction was temporarily stabilized with one anteriorly inserted 2.0 mm K-wire; a second pin was used to prevent reduction loss during fluoroscopy assessment. Final fixation was achieved by three or four 2.8 mm threaded pins, inserted through the lateral incision (Fig. 1.3). Rynning et al. showed that fixation with threaded pins was as stable as other fixation modalities.^26^ Epiphyseal vascularity was reassessed prior to layered closure. A direct view on pulsating cephalad branches or a continuous blood flow coming from a small drillhole in the epiphysis was considered as adequate vascular supply to the epiphysis. Following surgery, patients remained non-weight-bearing for six weeks, and the timing of hardware removal was guided by radiographic union.

**Table:** 

Surgical tips and tricks
• Use a curved osteotome • Remove physis (and fragment the posterior callus) • Use convex side of osteotome as fulcrum to lever the metaphysis posteroinferiorly into internal rotation • Hip flexion redirects the metaphysis in valgus • Remove superior metaphyseal bone to minimize traction on vessels • Use Schanz screw in greater trochanter for traction • Use repeated, gentle maneuvers • Be patient

### 2.4 Statistical analysis

Statistical analyses were performed using R (version 4.5.3).^27^ Descriptive statistics were used to summarize patient demographics and clinical characteristics. Continuous variables were presented as median and interquartile range (IQR), while categorical variables were reported as count and percentages.

The distribution of continuous variables was assessed visually and by means of the Shapiro-Wilk test. Given the small sample-size, non-parametric testing was preferred. Preoperative and postoperative Southwick angles were compared using the Wilcoxon signed-rank test for paired samples. A p-value <0.05 was considered statistically significant. Interobserver agreement of radiographic measurements between the two independent observers was assessed using the intraclass correlation coefficient (ICC). A two-way random-effects model with absolute agreement for single measurements was applied. Corresponding 95% confidence intervals were calculated. ICC values were interpreted as poor (<0.5), moderate (0.5–0.75), good (0.75–0.9), or excellent (>0.9).

## 3 Results

Open epiphyseal realignment through the DAA for SCFE was performed in 12 patients (13 hips; 8 left and 5 right) of which 9 were boys and 3 were girls (9 male hips, 4 female hips). Patient characteristics are shown in (Table 1). Median age at date of surgery was 12.9 years old (IQR 12.2-13.7) and median Body Mass Index (BMI) was 23.1 kg/m^2^ (IQR 19.9-29.5). Median follow-up was 6.0 years (IQR 2.4-8.3).

**Table 1:** Patients’ characteristics including clinical and radiographic evaluation.

Variable	Value (median, IQR)
Age (years)	12.9 (12.2–13.7)
BMI (kg/m^2^)	23.1 (19.9–29.5)
Male (n, %)	9, 69.2%
Stable (n, %)	8, 61.5%
Acute (n, %)	4, 30.8%
Traumatic (n, %)	5, 38.5%
Follow-up (y)	6.0 (2.4–8.3)
Alpha angle (°)	44.5 (42.8–68.1)
Hip length discrepancy (mm)	−5.4 (−8.3 to −0.76)
Pre-op AP Southwick	117.5 (95.8–137.3)
Post-op AP Southwick	139.5 (135.0–143.3)
Pre-op lateral Southwick	59.6 (35.1–71.8)
Post-op lateral Southwick	14.7 (11.3–20.9)
Correction lateral	31.2 (17.4–54.2)
Residual slip	4.7 (1.3–10.9)
AVN (n, %)	0, 0%
Hardware removal (n, %)	13, 100%
Surgical time (min)	85 (80–110)^a^
Length of hospital stay (hours)	54.5 (52–74.8)^b^

Abbreviations: BMI = Body Mass Index; AP = Anteroposterior; AVN = Avascular Necrosis; IQR = Interquartile Range. Values are reported per patient (n = 12) for Age, BMI, Sex and Follow-up, and per hip (n = 13) for all other variables.

a = 4 missing values.

b = 3 missing values.

Based on onset of complaints, four cases can be classified as acute -, two as acute-on-chronic and seven as chronic cases. A traumatic event was reported in five cases. In eight cases, weight bearing was still possible and could therefore be considered as stable. One of the patients had bilateral involvement. Prophylactic in-situ fixation of the contralateral hip was performed in three patients, due to onset of clinical complains. None of the patients developed superficial or deep wound infections. No cases developed failure of material requiring revision surgery.

Interobserver agreement was excellent for both AP and lateral Southwick angle measurements, as well as for the alpha angle and HLD at final follow-up, with ICC values ranging from 0.874 to 0.987 (Table 2). Median preoperative AP - and lateral Southwick angle were respectively 117.5 (IQR 95.8–137.3) and 59.6 (IQR 35.1–71.8) degrees. Based on these measurements, one patient was treated for mild -, five for moderate and seven for severe SCFE. Median postoperative AP – and lateral Southwick angles were respectively 139.5 (IQR 135.0–143.3) and 14.7 (IQR 11.3–20.9). Thus, a median correction of the lateral Southwick angle of 31.2 (IQR 17.4–54.2) was achieved. Individual pre-to postoperative changes in AP Southwick angle are illustrated in Fig. 2. Individual changes in lateral Southwick angle are shown in Fig. 3. Median Alpha-angle on Dunn view at final follow-up was 44.5 (IQR 42.8–68.1). Median HLD at final follow-up was −5.4 (IQR -8.3 to −0.76).

**Table 2:** Interobserver reliability of AP and lateral Southwick angle measurements.

Measurement	ICC	Lower CI	Upper CI
Preoperative AP Southwick angle	0.987	0.959	0.996
Postoperative AP Southwick angle	0.874	0.646	0.960
Preoperative lateral Southwick angle	0.972	0.913	0.991
Postoperative lateral Southwick angle	0.943	0.821	0.982
Alpha angle at final follow-up	0.887	0.681	0.964
Hip Length Discrepancy	0.985	0.945	0.996

Abbreviations: AP = Anteroposterior; ICC = Intraclass Correlation Coefficient; CI = Confidence Interval.

**Fig. 1:**
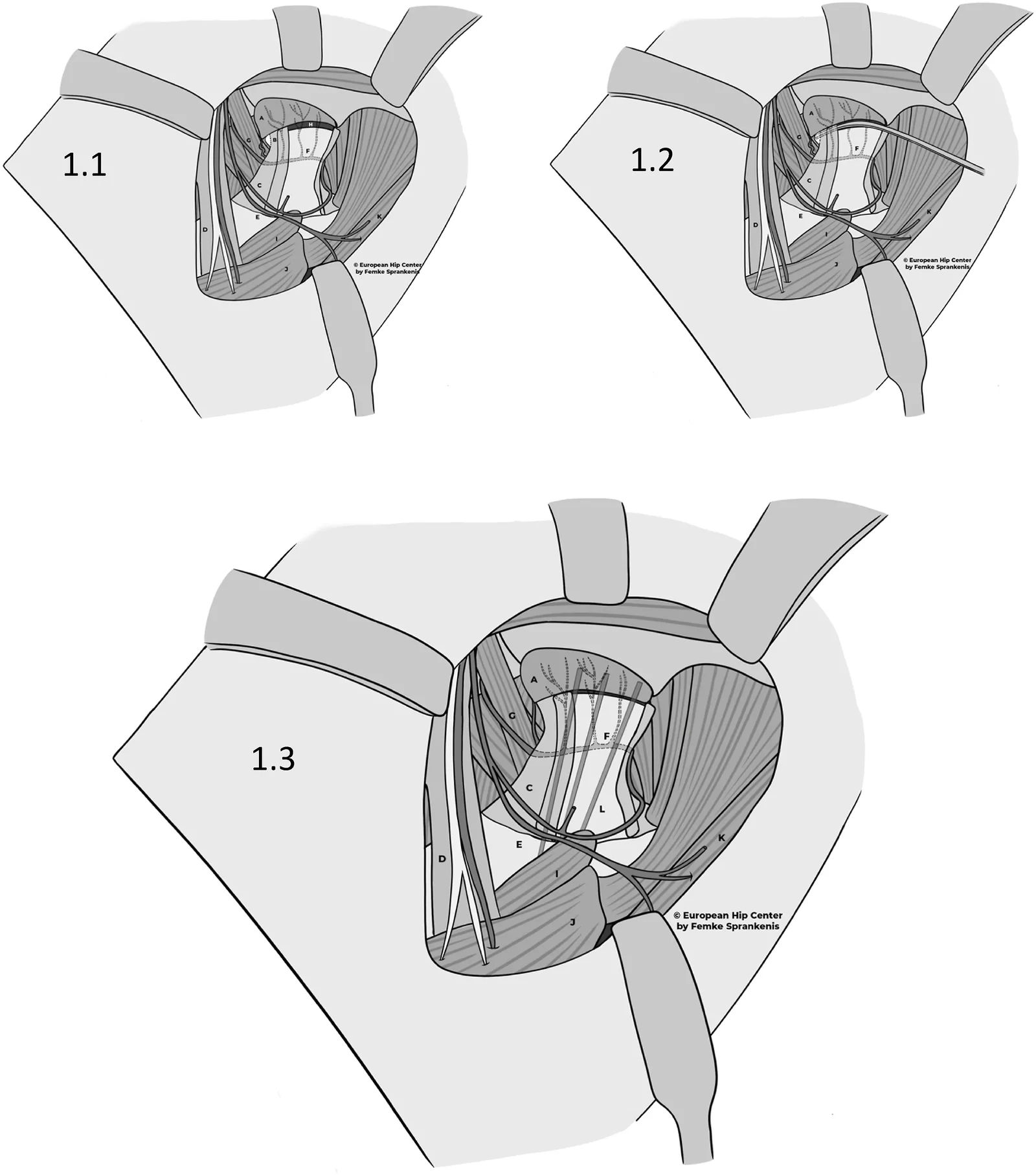
Schematic illustration of a left hip in the supine position exposed via a bikini-incision direct anterior approach, with the capsule omitted for clarity. **1.1.** The capital femoral epiphysis remains acetabular, whereas the metaphysis has slipped anteriorly and into external rotation across the physis. The epiphysis lies beyond the displaced metaphysis, leaving the posterior retinacular vessels relaxed rather than under tension. **1.2.** A curved osteotome was used to gradually open the physis with controlled taps, beginning centrally, followed by inferior and then superior extension. The physeal tissue was removed, and the posterior callus fragment was fragmented carefully under indirect tactile guidance. **1.3.** Final fixation was achieved by three or four 2.8 mm threaded pins, inserted through a lateral incision. A, femoral epiphysis; B, epiphysis beyond the displaced metaphysis; C, periosteal sleeve of the femoral neck; D, first neurovascular bundle; E, ascending branches to the tensor fasciae latae and anterior capsule; F, posterior retinacular branches; G, medial femoral circumflex artery; H, physis; I, vastus intermedius; J, vastus lateralis; K, tensor fasciae latae.

**Fig. 2:**
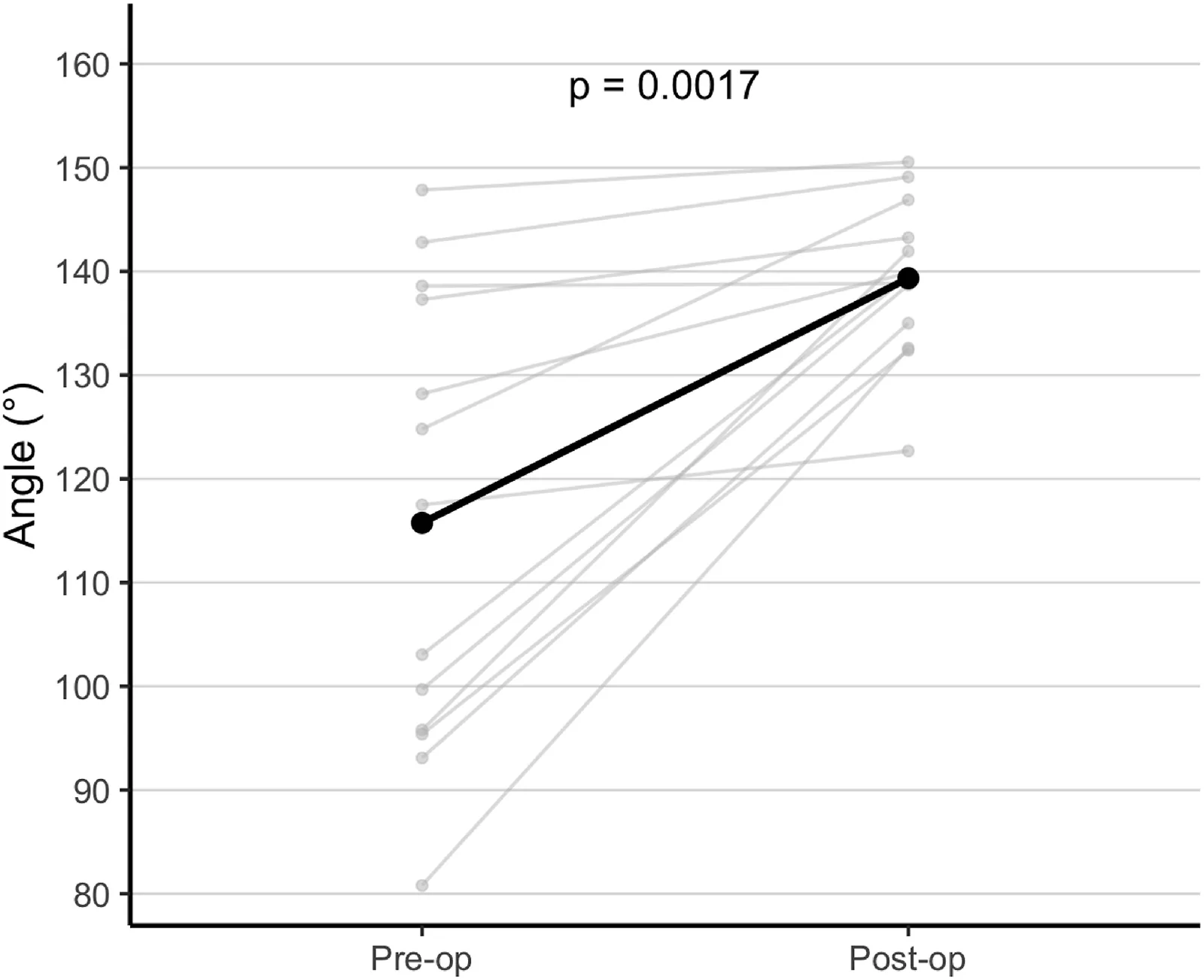
**Pre-to postoperative change in AP Southwick angle (n = 13 hips).** Paired dot plot showing the individual preoperative and postoperative AP Southwick angle for each of the 13 hips (grey dots, connected by grey lines). The bold black line connects the cohort median values (preoperative median 117.5°, IQR 95.8–137.3°; postoperative median 139.5°, IQR 135.0–143.3°). The AP Southwick angle increased in nearly all hips after open realignment (Wilcoxon signed-rank test, p = 0.0017).

**Fig. 3:**
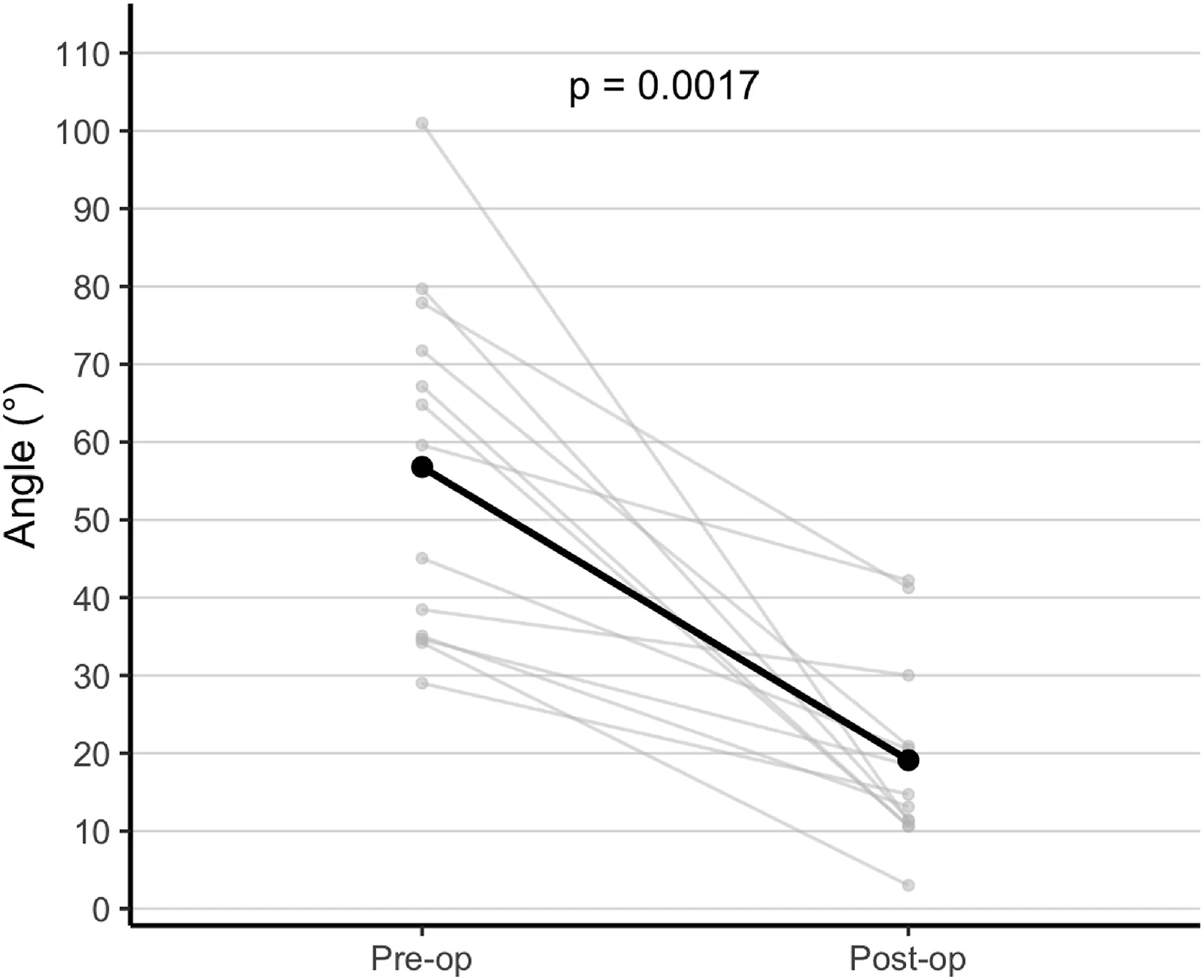
**Pre-to postoperative change in lateral Southwick angle (n = 13 hips).** Paired dot plot showing the individual preoperative and postoperative lateral Southwick angle for each of the 13 hips (grey dots, connected by grey lines). The bold black line connects the cohort median values (preoperative median 59.6°, IQR 35.1–71.8°; postoperative median 14.7°, IQR 11.3–20.9°). The lateral Southwick angle decreased (i.e., the slip was corrected) in all hips after open realignment (Wilcoxon signed-rank test, p = 0.0017).

**Fig. 4:**
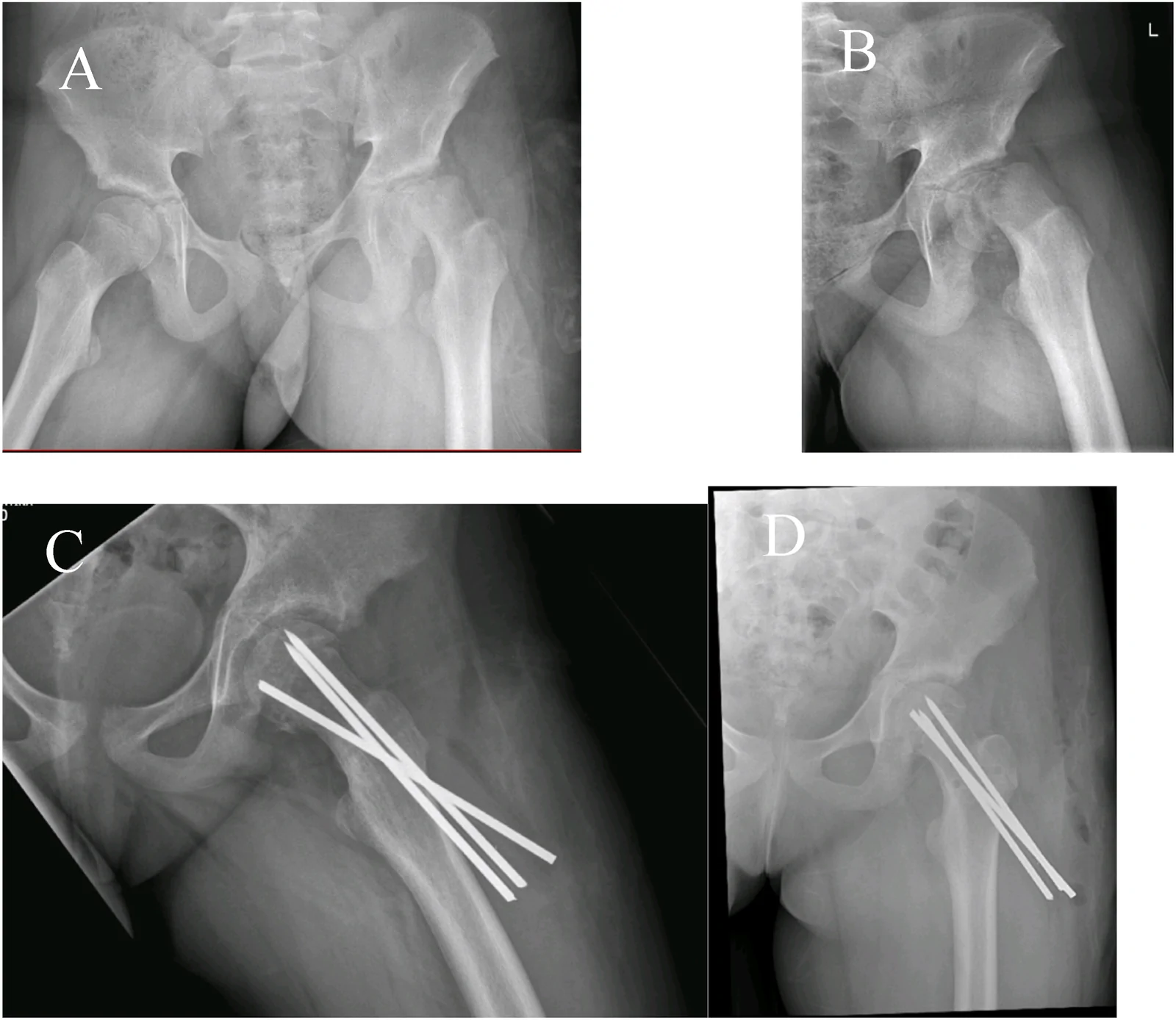
Radiographic images from (A and B) a severe SCFE of the left hip, treated by open realignment through DAA (C and D). Realignment fixation was conducted by three treaded K-wires, inserted from a lateral incision. The K-wires were left prone to ease removal after three months.

Radiographically union was confirmed in all cases by the 3-month follow-up visit. Removal of all hardware was executed at a median of 9.7 months (IQR 7.8–17.0). No patients developed heterotopic ossifications, chondrolysis or nonunion. One patient had an intra-articular positioning of the pin and required repositioning within 3 days after realignment. At final follow-up, a remnant of the posterior callus remained as a prominent bony bump in five patients.

Blood flow to the epiphysis was intraoperatively monitored and two patients showed absence of backflow.^28^ None of the patients developed radiological signs of avascular necrosis on any of the postoperative X-rays.

## 4 Discussion

Open realignment for SCFE through the historically extensile anterior Smith-Petersen approach was abandoned due to high postoperative AVN-rates.^28^ The Hueter-interval in DAA utilizes the same internervous and intermuscular plane as a less invasive and soft-tissue preserving alternative.^18^ The DAA can preserve the ascending branches of the LCFA and eliminates the need for trochanteric osteotomy. In all cases of our series, these vessels were intact at the end of the procedure.

The primary goal of open realignment is to achieve anatomic correction. The Bernese group reported residual slip angles between 4° and 8° for the Modified Dunn-technique.^15^ The anterolateral Parsch-technique achieved a mean correction of 32° with a residual slip angle of 10.6°.^19^ For DAA, Faldini et al. reported on SCFE correction through the Hueter interval in 9 patients with a mean follow-up of 28 months.^21^ The mean slip-angle corrected from 41° to 12°. None of the patients developed AVN.^21^ Niane et al. applied the anterior approach for SCFE correction in 26 hips (24 patients).^29^ Mean follow-up was 5 years. The mean preoperative slip-angle was 57° and a correction of 47° was achieved postoperatively. Because of suboptimal realignment, five patients went on to developed chondrolysis and one osteo-necrosis.^29^ In our cohort, correction of the lateral Southwick angle was 31.2° (IQR 17.4–54.2) with a residual slip angle of 4.7° (IQR 1.3-10.9). These outcomes are in line with the good results reported by the Bernese center (2017), Parsch et al. (2009) and Faldini.^15^^,^^19^^,^^21^ These findings support that SCFE realignment through the DAA is feasible and does not appear to limit correction potential (see Fig. 4).

In five patients, a persistent bony posterior callus prominence was observed, most likely reflecting incomplete resection of the posterior callus because of the limited posterior acces by the DAA. This represents an important technical limitation of the approach, particularly in chronic and severe slips, where posterior callus formation may be more pronounced. Residual posterior deformity may theoretically contribute to persistant posterior femoroacetabular impingement and, over time, secondary degenerative changes. However, in the present cohort, only one patient required subsequent hip arthroscopy for labral repair three years after open realignment. Also, HLD was -5.4 (IQR -8.3 to -0.76). Given that the physis was removed and only three of the patients underwent contralateral prophylactic in situ pinning, this finding aligns with the anticipated consequence. However, non of the patients had restrictions in daily life activities and all were able to perform recreative sports as running, soccer, tennis or biking. Therefore, the clinical relevance of these findings remains unclear. However, no validated patient-reported outcome measures (e.g., Harris Hip Score, iHOT-12, or WOMAC) were systematically collected, which limits objective assessment of functional outcome.

However, none of the patients had restrictions in daily life activities and all were able to perform recreative sports as running, soccer, tennis, or biking. Therefore, the clinical relevance of these findings remains unclear. However, no validated patient-reported outcome measures (e.g., Harris Hip Score, iHOT-12, or WOMAC) were systematically collected, which limits objective assessment of functional outcome.

In our series of 13 SCFE-hips, no cases of AVN occurred. In current literature, reported AVN-rates following open realignment vary widely. A low incidence is seen when using the modified Dunn procedure with an AVN rate of 2% across two series of 30 and 23 patients.^21^^,^^30^ Also, Ziebarth et al. (2009) reported no cases of AVN in 40 patients from two centers with a follow-up of 5.4 and 2.2 years.^11^ Conversely, a high AVN-rate of 25% with follow-up ranging 2-20 years was reported by Valenza et al. (2022).^31^ In addition, de Moura Vallim et al. (2022) reported a rate of 26.7% in 30 patients with a follow-up of 3.1 years.^32^ Meta-analysis further illustrates this variability: Cheok et al. (2022) reported an odds ratio of 18.5% for AVN and chondrolysis.^17^ Veramuthu et al. (2022) identified a pooled AVN prevalence of 19.9% across 33 studies.^22^ Using an anterolateral approach, Parsch et al. (2009) described an open realignment without direct visualization of the epiphysis, slip correction, or real-time vascular assessment. Three of their 64 patients developed AVN postoperatively.^19^

This study has several limitations. *First*, it constitutes a retrospective case-series with a small sample size and lacks a matched control group treated with the modified Dunn procedure. Therefore, it should be regarded as a pilot-study describing an alternative approach for open realignment of stable and unstable SCFE. *Second*, direct visualization of the posterior periosteal flap, containing branches of the deep division of the medial circumflex femoral artery (MCFA), is limited with the DAA. Therefore, intraoperative assessment of epiphyseal perfusion is primarily based on backflow evaluation.^33^ In addition, an unresected posterior callus leads to a posterior bump in chronic and acute-on-chronic cases. *Third*, as this study is a retrospective case-series spanning an 11-year inclusion period (January 2014 to January 2025), intraoperative blood loss was not systematically or uniformly documented in the electronic patient records and could therefore not be reliably reported for the whole cohort. Likewise, validated functional or patient-reported outcome measures (e.g., Harris Hip Score, iHOT-12, WOMAC) were not systematically collected. We recommend that future prospective studies of this technique incorporate standardized recording of blood loss and validated functional/PROM scores, to allow direct comparison with the modified Dunn procedure literature. *Finally*, radiographic hip length discrepancy was measured on standard anteroposterior pelvic radiographs rather than on calibrated standing long-leg radiographs. Therefore, this measurement reflects a proximal radiographic estimate rather than true whole-limb length discrepancy and may be influenced by pelvic tilt, rotation, hip position, and the absence of consistent magnification correction.

## 5 Conclusion

The DAA described in this series, can be considered a reproducible technique for an open realignment of displaced SCFE. None of the cases had an AVN and the approach does not appear to limit correction potential.

## Patient consent

No patients consent needed for this type of study.

## Author credit statement

Frans-Jozef Vandeputte MD: Conceptualization, Methodology, Investigation, Surgery, Data curation, Writing – original draft, Writing – review & editing.

Siebe Clarebout, MD: Conceptualization, Methodology, Data curation, Writing – original draft,

Abner Sergooris, PhD: Methodology, Data Analysis, Writing – review & editing.

Mathieu Gevers MD: Conceptualization, Writing – original draft.

Frank Van Praet MD: Writing – original draft.

Kristoff Corten MD, PhD: Conceptualization, Methodology, Investigation, Surgery.

## Ethical approval

Ethical approval for this study was obtained from the institutional review board at Ziekenhuis Oost-Limburg (ZOL) Genk, Belgium (protocol number IRB-Z-2025128).

## Funding statement

The authors received no financial support for the research, authorship, and/or publication of this article.

## Declaration of competing interest statement

The authors declare that they have no known competing financial interests or personal relationships that could have appeared to influence the work reported in this paper.
